# Community Structure Analysis of Gene Interaction Networks in Duchenne Muscular Dystrophy

**DOI:** 10.1371/journal.pone.0067237

**Published:** 2013-06-19

**Authors:** Tejaswini Narayanan, Shankar Subramaniam

**Affiliations:** 1 Department of Electrical and Computer Engineering, University of California San Diego, La Jolla, California, United States of America; 2 Department of Bioengineering, University of California San Diego, La Jolla, California, United States of America; Goethe University, Germany

## Abstract

Duchenne Muscular Dystrophy (DMD) is an important pathology associated with the human skeletal muscle and has been studied extensively. Gene expression measurements on skeletal muscle of patients afflicted with DMD provides the opportunity to understand the underlying mechanisms that lead to the pathology. Community structure analysis is a useful computational technique for understanding and modeling genetic interaction networks. In this paper, we leverage this technique in combination with gene expression measurements from normal and DMD patient skeletal muscle tissue to study the structure of genetic interactions in the context of DMD. We define a novel framework for transforming a raw dataset of gene expression measurements into an interaction network, and subsequently apply algorithms for community structure analysis for the extraction of topological communities. The emergent communities are analyzed from a biological standpoint in terms of their constituent biological pathways, and an interpretation that draws correlations between functional and structural organization of the genetic interactions is presented. We also compare these communities and associated functions in pathology against those in normal human skeletal muscle. In particular, differential enhancements are observed in the following pathways between pathological and normal cases: Metabolic, Focal adhesion, Regulation of actin cytoskeleton and Cell adhesion, and implication of these mechanisms are supported by prior work. Furthermore, our study also includes a gene-level analysis to identify genes that are involved in the coupling between the pathways of interest. We believe that our results serve to highlight important distinguishing features in the structural/functional organization of constituent biological pathways, as it relates to normal and DMD cases, and provide the mechanistic basis for further biological investigations into specific pathways differently regulated between normal and DMD patients. These findings have the potential to serve as fertile ground for therapeutic applications involving targeted drug development for DMD.

## Background

Community structure analysis is an interesting computational technique for studying interaction networks. Analysis of community structure in networks can yield useful insights into the structural organization of the network. For instance, community structure analysis is used in the context of networks that arise in domains such as social networks to understand the fundamental social structure in a community of interacting individuals [1]–[7]. This provides insights about the influential individuals and the strongly-networked individuals in a community. Another domain where algorithms for community structure analysis find useful application is the topological understanding of large scale connection networks such as Internet, and how one may use the insights from community structure analysis to design more resilient communication networks [6], [8]–[10]. In the context of biological networks, such insights can also be used to understand the biological significance of the underlying community structure and organization of the network. There is existing work that discusses the use of community structure analysis in networks that are observed in biological contexts [4]–[5], [11]–[13]. For example, [4] presents the application of an algorithm for community structure analysis to a food web of marine organisms living in the Chesapeake Bay, a large estuary on the east coast of the United States. Furthermore, rich toolsets have also been developed for the purpose of understanding biological networks from a community structure perspective [13]–[17].

In this paper, we explore the application of community structure analysis as an effective technique to understand the topological structure and biological behavior of human skeletal muscle. Skeletal muscles are a form of striated muscle tissue existing under the control of the somatic nervous system, which are attached to bones by tendons. This muscle category has been clinically associated with diseases such as Myopathy, Muscular Dystrophy, Paralysis, and a host of other diseases. DMD is a group of inherited disorders that involve muscle weakness and loss of muscle tissue, which get worse over time [18] and results in death before the individual reaches adulthood. Given the genetic nature of this disorder, techniques that leverage the underlying genetic interactions are expected to yield useful insights, and this is the primary focus of our study.

### Community structure analysis: Newman and Girvan Algorithm

The Newman and Girvan (NG) algorithm is a popular algorithm for community structure analysis in networks [7]. It is a divisive approach that selects and removes edges based on its *betweenness* value. The betweenness of an edge is defined as the number of shortest paths between all vertex pairs in the network, which run along that edge. The steps involved in the NG algorithm are as follows: The betweenness values of all edges are computed. The edge with the largest betweenness is removed (in case of ties with other edges, one of them is picked at random). This is followed by the recalculation of betweenness values of the remaining edges in the network. The entire process is repeated iteratively till all edges are removed.

The output from this algorithm is a dendrogram capturing the possible division of the network into communities. In order to select the *optimal* split from these possible candidates, Newman and Girvan introduce the concept of *modularity*, which is a measure of the quality of a particular division of a network into communities [7]. Given a specific division of a network into 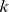 communities, let us define a 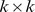
symmetric matrix 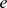 whose element 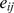 is the fraction of all edges in the original network that link vertices in community 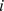 to vertices in community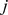. The row (or column) sums 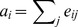 represent the fraction of edges that connect to vertices in community 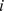. *Modularity* is then defined as follows:
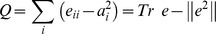



where 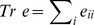, denotes the trace of the matrix 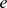 and 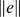 denotes the sum of the elements of the matrix 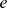. Typically, 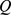 is calculated for each split of a network into communities as the algorithm moves down the dendrogram, with the optimal split corresponding to the peak value of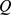. For a network with 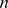 vertices and 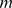 edges, the worst-case time complexity for this algorithm is 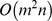 (or 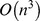 for a sparse network).

## Results and Discussion

Consequence of DMD pathology manifests in the state of muscle cells. The physiological state and cellular state of muscles are altered, involving concomitant changes in the expression of genes associated with the physiological function. In particular, gene expressions in DMD patients have the potential to provide information on distinguishing characteristics of pathology, relative to normal muscle (since altered gene expressions could aid in identification of functional communities). In this work, we have devised a novel approach to analyze human DMD patient gene expression data using a combination of techniques from linear algebra and network theory. Specifically, we posit that the correlation of gene expression data from DMD patients captures salient characteristics of pathology. Accordingly, we build the correlation network from the gene expression data for the normal and DMD muscles. Under the assumption that correlation implies mechanistic causality, we take the approach of community structure analysis, to identify functional communities from the correlation network, to display known functional and pathway mechanisms.

### Derived Interaction Networks

In this section, we present an analysis of the global properties of the *derived interaction networks* (defined in the Methods section) for the normal and DMD muscle data from a descriptive statistics standpoint. We use well known global network properties such as density, average degree etc. to inform our analysis. This analysis aims to highlight the key similarities and differences between the derived interaction networks for normal and DMD muscle data, in order to enable a structural understanding of the underlying genetic interactions at a macro level.

**Figure 1:**
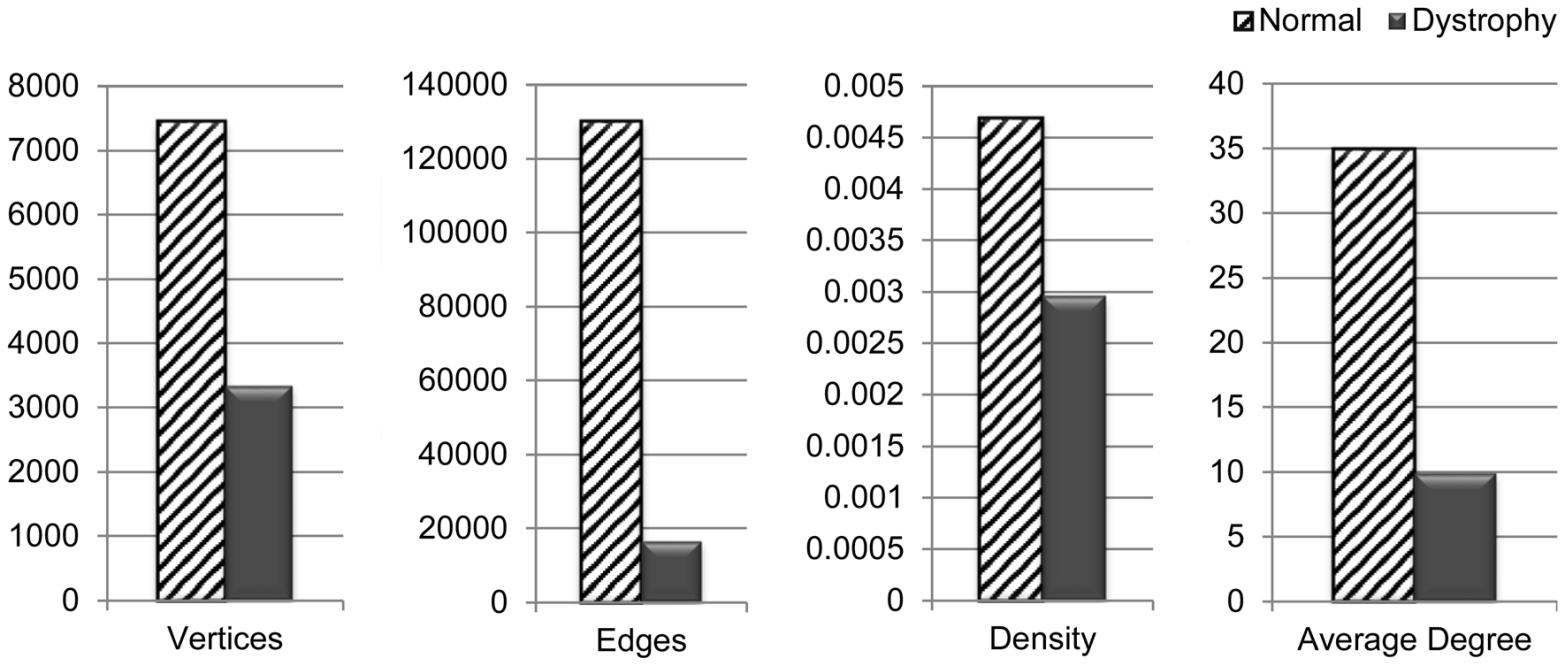
Structural Properties- Normal vs. Dystrophy Interaction Networks. Plots of the number of Vertices, number of Edges, Density and Average Degree of the Normal and DMD interaction networks that were constructed from the GSE6011 dataset [discussed in Methods Section]. The scales (y-axis) for these structural properties are different and the data for the networks are color coded as green and red for the Normal and DMD muscles respectively.

Figure 1 illustrates the key structural differences in the normal and the DMD interaction networks. As can be noted from Table 1, the number of vertices and edges in the DMD interaction network is much smaller than those of the normal interaction network. Thus, as one would expect, the density and the average degree of the DMD interaction network are also lesser than the normal network (as shown in Figure 1). However, it is interesting to note that *both* interaction networks have turned out to be *sparse* from a network-theoretic standpoint.

**Table 1:** Summary of interaction networks for normal and DMD muscle.

Dataset	Number of vertices considered*	Number of edges
Normal muscle	7453	130225
DMD muscle	3332	16445

* The original number of vertices after pre-processing the GSE6011 dataset was 7685.

From the planar-layout visualization of the normal and the DMD interaction networks generated using Cytoscape [19], we observe that the pre-processed networks containing 7685 vertices are by themselves disconnected into many independently connected components. Table 2 summarizes the key network parameters for the normal and DMD cases for the whole interaction map.

**Table 2:** Summary of network parameters.

Full Network	Normal	DMD
Vertices	7453	3332
Edges	130225	16445
Clusters	670	283
Clustering coefficient	0.278	0.216
Connected components	27	219
Network diameter	16	24
Network radius	1	1
Network centralization	0.056	0.052
Shortest paths	98%	71%
Characteristic path length	5.302	7.852
Avg. number of neighbors	34.946	9.871
Network density	0.005	0.003
Network heterogeneity	1.819	2.042

Since we are interested in finding communities from the networks, we consider the largest connected component in both networks. Table 3 shows the number of vertices and edges considered for community structure analysis in both the networks (i.e. the parameters defining the largest components in the respective interaction networks).

**Table 3:** Parameters of networks' largest component used for community structure analysis.

Dataset	Number of vertices	Number of edges
Normal muscle	7389	130185
DMD muscle	2823	16142

### Community structure analysis

**Figure 2:**
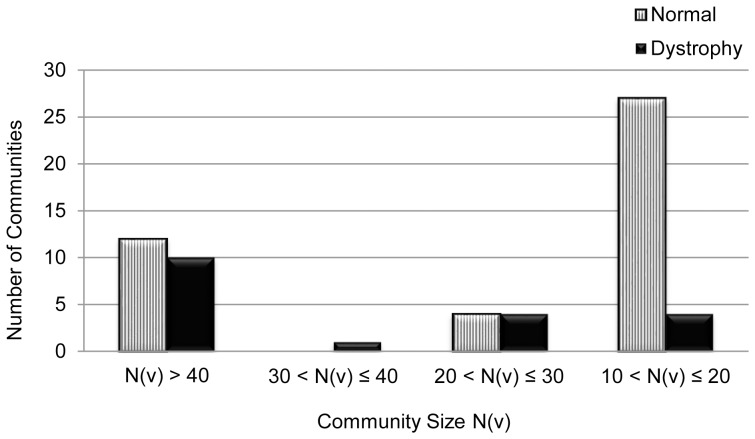
Distribution of Communities. A comparison of the distribution of communities in both the Normal and DMD networks, obtained using the Newman and Girvan's edge-betweenness algorithm. The green bars show the distribution of the total number of 644 communities obtained from the Normal network, across the four bins of community size, and the red bars represent the distribution of the 283 communities from the DMD network.

In this section, we present our results from running the NG algorithm on the largest components of the derived interaction networks for the normal and DMD muscle datasets. Table 4 presents the number of communities identified in the dataset, along with the corresponding modularity values (Q_max_). We provide in Figure 2, a comparison of the distribution of communities in both networks (obtained using the NG algorithm), across bins defined by vertex cardinality range.

**Table 4:** Communities from the GSE6011 dataset.

Dataset	Number of communities	Q_max_
Normal muscle	670	0.498339
DMD muscle	283	0.535499

### Pathway Analysis

We perform an analysis of the communities obtained from the NG algorithm from the perspective of its constituent pathways, by generating *pathway projection networks* (PPNs). The motivation, technique and color-coding convention of PPNs are detailed in the Methods section.

**Figure 3:**
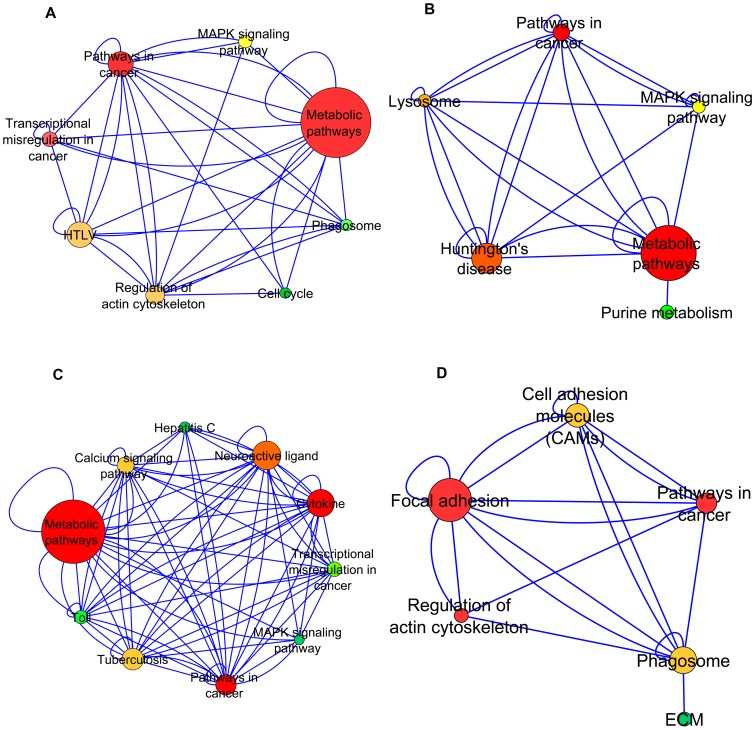
Pathway Projection Networks. Representation of communities (of interest) from the perspective of the pathways. Nodes in the PPNs are derived from (and are representative of) the pathway(s) that the constituent genes correspond to. The edges between the pathway-nodes represent the connections between the underlying genes in the original network. The nodes are color-coded according to the degree (measure of connectivity between the pathways) and size-coded according to the pathway cardinality of the node (number of genes from the community that correspond to that pathway). The transformation technique that was employed to generate an equivalent network in terms of the constituent pathways for each community is described in the Methods section [also schematically presented in the flowchart in Figure 4].

Figure 3 illustrates the PPNs that are considered for analysis.

### Biological Interpretation and Discussion

While we have included a representative set of PPNs in the Supporting Information (Figure S1- Figure S11), we consider the 4 PPNs shown in Figure 3 to elucidate the significance of the pathways of interest (shown in Table 5) in each community and the correlation to their presence in the PPNs. Specifically, the pathways we consider are Metabolic, Focal adhesion, Regulation of actin cytoskeleton and Cell adhesion. We are interested in finding evidence from past work that can potentially help with triangulating our algorithmic findings about the specific pathway enhancements that we have identified. For example, if a specific pathway is determined to be enhanced by our algorithmic technique, we would expect the evidence corresponding to that pathway to correlate well with such an enhancement (for the network under consideration). Conversely, for a pathway that is determined to not have a pronounced enhancement using our algorithmic approach, we are interested in finding whether the experimental evidence surrounding that pathway is aligned with our finding. We believe that this analysis will help us validate our algorithmic findings with evidence from existing research. We also perform a gene-level analysis on the PPNs to identify genes that are involved in the coupling between the corresponding pathways of interest, and summarize sample gene pairs with their corresponding correlation scores. We leverage UniProtKB [20] for identifying the functional information associated with the sample genes we consider in the discussion below.

**Table 5:** Pathways of interest in each community.

Pathway projection network	Pathway of interest
PPN1–PPN3	Metabolic pathways
PPN4	Focal adhesion, Regulation of actin cytoskeleton and Cell adhesion molecules

As background for rest of the discussion, we note that *dystrophin* is a key protein of interest in the study of dystrophy. Specifically, the absence of *dystrophin* is associated with DMD and was identified as the source of pathology in humans using positional cloning [21]. Mice lacking dystrophin have high serum levels of muscle enzymes and possess histological lesions similar to human muscular dystrophy [22]–[24].

### Metabolic pathways and DMD

Our results emphasize an interesting connection between metabolic pathways and DMD, and we leverage Figures 3A–3C (PPN 1-PPN 3) to explore these connections in greater detail. We summarize the key observations from our analysis here. The first observation from Figures 3A–3C is that, PPN 1–PPN 3 exhibit enhanced representation of metabolic pathways. Furthermore, Figure 3A (PPN 1) illustrates a strong coupling between metabolic pathways and regulation of actin cytoskeleton. Similarly, we observe a direct coupling of metabolic pathways to calcium signaling from Figure 3C. Finally, we reiterate the importance of metabolism as a key differentiator in pathology, in terms of glycolytic and oxidative variations of metabolic pathways. The rest of this section provides evidence from prior work in this domain to support our observations.

Our first observation around metabolic pathways and their connection to DMD, is in alignment with prior work. In particular, [22] identifies that a dystrophin-dependent cytoskeletal organization in skeletal muscles is directly related to the efficiency of cytoplasmic and mitochondrial metabolic pathways in situ. More generally, the lack of dystrophin or a functionally mildly defective dystrophin is connected with subnormal rates of muscle energy conversion and the subnormal energy status of sarcoplasm. In other words, enhancement of metabolic pathways is a canonical characteristic in normal muscle, and our findings (Figure 3A–3C) are consistent with this result. Also, from a computational standpoint, the observed specificity in enhancement validates the algorithm for community structure analysis used in our approach, since the algorithm grouped the genes corresponding to metabolic pathways in cohesive communities. Furthermore, a similar exercise of pathway projection performed on the DMD network had no significant representation of the metabolic pathways.

Secondly, the observation of strong coupling between metabolic pathways and regulation of actin cytoskeleton is corroborated by prior experimental work, which has identified that, muscles from the dystrophic *mdx* mouse show reduced maintenance metabolic rates [22]. The authors of [22] also propose that the in vivo efficiency of metabolic pathways may depend on stabilization of enzyme complexes by dystrophin-associated elements of the cytoskeleton. By performing a gene-level analysis on PPN 1 (Figure 3A), we found that many genes were involved in the coupling between the two pathways of interest. Table 6 presents five sample gene pairs and the corresponding correlation scores between them.

**Table 6:** Sample correlation scores of highly correlated genes (Metabolic and Regulation of actin cytoskeleton pathways).

Highly correlated genes		Correlation Score
Metabolic pathway	Regulation of actin cytoskeleton	
Leukotriene A4 hydrolase	Cell division cycle 42	0.876235649
Phosphoinositide-3-kinase, class 2, alpha polypeptide	Platelet-derived growth factor receptor, alpha polypeptide	0.870928383
Phosphoglycerate mutase 1	Cofilin 1	0.860115666
Iduronate 2-sulfatase	integrin, alpha V	0.849397619
dCMP deaminase	Actinin, alpha 4	0.82944117

Specifically, Leukotriene A4 hydrolase is an epoxide hydrolase that catalyzes the final step in the biosynthesis of the proinflammatory mediator leukotriene B4 [20]. This gene is highly correlated with cell division cycle 42 which is involved in epithelial cell polarization processes. It also plays a role in the extension and maintenance of the formation of thin, actin-rich surface projections called filopodia. Phosphoglycerate mutase 1 is highly correlated with Cofilin 1 which regulates actin cytoskeleton dynamics and plays a role in the regulation of cell morphology [20]. It is interesting to note that a similar correlation was observed between these genes in astrocytomas involved in pathogenesis of radioresistance [25]. There is existing evidence of association between Iduronate 2-sulfatase and integrin, alpha V from a Gene Set Enrichment Analysis point of view (which is in accordance with the results shown in Table 6, in terms of their correlation) [26]. Iduronate 2-sulfatase plays a role in the lysosomal degradation of heparan sulfate and dermatan sulfate. integrin, alpha V is a receptor for fibronectin and fibrinogen [20]. Finally, referring to the high correlation between PIK3CA and PDGFRB, there is existing evidence that reports an interaction between these genes [27].

Similarly, we note that there is evidence from past research that aligns with our observation around the coupling of metabolic pathways to calcium signaling. In particular, [28] suggests that high intracellular Ca 2+ (linked to calcium signaling) in dystrophic fibers, may be the cause of the inefficiency of mitochondrial metabolic pathways. Table 7 provides five sample gene pairs with their corresponding correlation scores, from among the many genes that we found to be highly correlated in function between the metabolic and calcium signaling pathways.

**Table 7:** Sample correlation scores of highly correlated genes (Metabolic and Calcium signaling pathways).

Highly correlated genes		Correlation Score
Metabolic pathway	Calcium signaling pathway	
Cytochrome P450, family 2, subfamily B, polypeptide 6	Phosphodiesterase 1C, calmodulin-dependent 70 kDa	0.970659478
Cytochrome P450, family 2, subfamily C, polypeptide 9	Phosphodiesterase 1C, calmodulin-dependent 70 kDa	0.945775382
Gamma-glutamyltransferase 1	Calcium/calmodulin-dependent protein kinase IV	0.912367742
Cysteine conjugate-beta lyase, cytoplasmic	Phosphodiesterase 1C, calmodulin-dependent 70 kDa	0.906362395
Fructose-1,6-bisphosphatase 1	v-erb-a erythroblastic leukemia viral oncogene homolog 4	0.900885014

While CYP2C6 plays a role in drug metabolism [29], CYP2C9 localizes to the endoplasmic reticulum and its expression is induced by rifampin. From Table 7, we observe that both CYP2C6 and CYP2C9 are highly correlated to Phosphodiesterase 1C, calmodulin-dependent 70kDa. Members of the Cyclic nucleotide phosphodiesterases (PDE1) family, are calmodulin-dependent PDEs [CaM-PDEs] that are stimulated by a calcium-calmodulin complex [30]. This gene is also highly correlated to Cysteine conjugate-beta lyase, cytoplasmic (from Table 7). ErbB-4 protein binds to and is activated by neuregulins and induces a variety of cellular responses including mitogenesis and differentiation [20]. It is interesting to note that this gene is highly correlated to Fructose-1,6-bisphosphatase 1, deficiency of which is associated with hypoglycemia and metabolic acidosis [31].

Analysis of functional communities that are differentially regulated, demonstrates metabolism as the most important mechanistic change in DMD muscle. In particular, glycolysis and oxidative metabolism play significant roles in muscle energetics including remodeling of the muscle into fast and slow fiber forms responding to the nature of the energy demands. Experiments that have been performed on normal muscle showed accumulation of glycolytic and oxidative metabolism capacity with increased age, but this accumulation failed in DMD [32]. The data used in [32] shows stage-specific remodeling of human dystrophin-deficient muscle, with inflammatory pathways predominating in the presymptomatic stages and failure of metabolic pathways later in the disease [32]–[33].

In the slow twitch (type I) fibers, the slow muscles are more efficient at using oxygen to generate more fuel (known as ATP) for continuous, extended muscle contractions over a long time. In other words, these are the fibers that correspond to oxidative phosphorylation. Whereas, because fast twitch (Type II) fibers use anaerobic metabolism to create fuel, they are much better at generating short bursts of strength or speed than slow muscles. These typically correspond to glycolysis/gluconeogenesis, which is involved in converting glucose into pyruvate. We performed an analysis on the number of genes that contributed to the fast and slow twitch fibers, in the three communities in which metabolic pathways were enhanced (PPN 1–PPN 3). The results are summarized in Table 8.

**Table 8:** Summary of muscle fibers' cardinality.

ID	Pathway	PPN1 (Fig 3A)	PPN2 (Fig 3B)	PPN3 (Fig 3C)
**hsa00010**	Glycolysis/Gluconeogenesis (fast twitch)	4	1	5
**hsa00190**	Oxidative phosphorylation (slow twitch)	18	13	6

### Regulation of actin cytoskeleton and DMD

The discussion on Regulation of actin cytoskeleton and its relationship to DMD is centered around Figure 3D (PPN 4). Specifically, PPN 4 illustrates that in normal skeletal muscle, the actin cytoskeleton pathways are enhanced, whereas they are less utilized in DMD muscle. This is consistent with prior work as follows. Dystrophin links the actin cytoskeleton to the dystroglycan complex (which is a part of an adhesion receptor complex [34]) in the plasma membrane as part of the linkage between the cytoskeleton and the extracellular matrix [35]–[36]. This link helps maintain sarcolemmal integrity in a muscle [37]. Damage to or absence of or mutations in dystrophin causes DMD [21], [36]–[37].

The skeletal muscle L-type Ca^2+^ channel (Ca_V_1.1), which is responsible for initiating muscle contraction, is regulated by phosphorylation by cAMP-dependent protein kinase (PKA) in a voltage-dependent manner [38]. Furthermore, the role of the actin cytoskeleton in channel regulation was investigated in skeletal myocytes cultured from *mdx* mice that lack the cytoskeletal linkage protein dystrophin, and a skeletal muscle cell line, 129 CB_3_. Results of the experiments detailed in [38] show that regulation of Ca^2+^ channel activity by hormones and neurotransmitters that use the PKA signal transduction pathway may interact in a critical way with the cytoskeleton and may be impaired by deletion of dystrophin, contributing to abnormal regulation of intracellular calcium concentrations in dystrophic muscle.

We see that most pathways in PPN4 are well-coupled to each other. From the sample correlation scores provided in Table 9, we infer that there is strong correlation [39] that exists between the genes, which signifies the coupling between the regulation of actin cytoskeleton and focal adhesion pathways.

**Table 9:** Sample correlation scores of highly correlated genes (Focal adhesion and Regulation of actin cytoskeleton pathways).

**Highly correlated genes**		**Correlation Score**
**Focal adhesion pathway**	**Regulation of actin cytoskeleton**	
Kinase insert domain receptor (a type III receptor tyrosine kinase)	Moesin	0.930732193
Laminin, alpha 4	Integrin, alpha 6	0.916349568
Collagen, type IV, alpha 2	Actin, beta	0.914346045
Collagen, type IV, alpha 1	Actin, beta	0.910817128
Laminin, alpha 4	Actin, beta	0.9039736

### Focal adhesion and DMD

We use Figure 3D (PPN 4) to motivate the discussion around the focal adhesion pathway, and its relationship to pathology. In particular, PPN 4 shows the expected level of association of focal adhesion pathways in normal muscle and this is consistent with the evidence presented below. The representation of focal adhesion kinase (FAK) in dystrophy networks has been studied previously [23], [40]. For example, the authors of [40] find that at 12 weeks of age, both hind limb muscles of dystrophic mice possessed a lower FAK protein than normal mice. It is proposed that FAK is a part of the pathway that would be of potential importance in transducing mechanical signals from cell membranes to skeletal muscle fiber nuclei [41]–[42]. Focal adhesion pathway is coupled tightly not only to regulation of actin cytoskeleton (as shown in the Table 9), but also to cell adhesion molecules, with high correlation scores, some of which are shown in Table 10.

**Table 10:** Sample correlation scores of highly correlated genes (Focal adhesion and Cell adhesion molecules pathways).

Highly correlated Genes		Correlation Score
Focal adhesion pathway	Cell adhesion molecules (CAMs)	
Laminin, alpha 4	Platelet/endothelial cell adhesion molecule 1	0.964950625
Laminin, alpha 4	Cadherin 5, type 2 (vascular endothelium)	0.939171386
Collagen, type IV, alpha 1	Platelet/endothelial cell adhesion molecule 1	0.937020588
Integrin, alpha 6	Cadherin 5, type 2 (vascular endothelium)	0.929446836
Actin, beta	Platelet/endothelial cell adhesion molecule 1	0.929072591

Referring to genes in Table 9, Laminin alpha-4 is a protein thought to mediate the attachment, migration and organization of cells into tissues by interacting with other extracellular matrix components, by binding to cells via a high affinity receptor [20]. Integrin alpha-6 is a receptor for laminin in epithelial cells and it plays a critical structural role in the hemidesmosome. Laminin alpha4 and integrin alpha6 are upregulated in regenerating dy/dy skeletal muscle [20]. Furthermore, laminin alpha4 and integrin alpha6 expression patterns are notably different in dy/dy when compared to normal muscle. This is especially pronounced in the interstitium of regenerating areas and on newly formed myotubes [43]. Our observation about the high correlation between Laminin alpha4 and integrin alpha6 (Table 9) is in alignment with these findings.

We also present a brief description (collated from [20]) of other genes in Table 9 amongst which we observe a high correlation. Moesin is conjectured to be involved in connections of major cytoskeletal structures to the plasma membrane. Kinase insert domain receptor (a type III receptor tyrosine kinase) is a vascular endothelial growth factor (VEGF) receptor. Beta-actin is one of six different actin isoforms which have been identified in humans. This is one of the two nonmuscle cytoskeletal actins. Actins are highly conserved proteins that are involved in cell motility, structure and integrity. Type IV collagen is the major structural component of glomerular basement membranes, forming a ‘chicken-wire’ meshwork together with laminins, proteoglycans and entactin/nidogen.

From Table 10, we observe that Platelet/endothelial cell adhesion molecule 1 (PECAM-1) and Cadherin 5, type 2 (vascular endothelium) genes from cell adhesion molecules pathway are highly correlated to the genes from the focal adhesion pathway. PECAM-1 is a transmembrane protein in the inter-endothelial cell contacts [20]. PECAM-1 is a homophilic adhesive molecule that is diffusely distributed on subconfluently growing endothelial cells, but concentrates at cell-cell borders upon cell-cell contact [44]. Our observation of high correlation between PECAM-1 and genes in the focal adhesion pathway (shown in Table 10) is corroborated by [45] which illustrates the co-localisation of some of the ECM components viz. laminin α1, collagen type IV with the endothelial cell marker PECAM-1. Cadherin 5, type 2 (vascular endothelium) are calcium-dependent cell adhesion proteins. They play an important role in endothelial cell biology through control of the cohesion and organization of the intercellular junctions [20]. From Table 10, we see that it is highly correlated with Integrin, alpha 6 and Laminin, alpha 4.

### Cell adhesion and DMD

Figure 3D (PPN 4) illustrates that the cell adhesion pathway is not enhanced significantly in the normal network (given that it is a relatively small sized node, representing smaller pathway cardinality). When we performed a detailed analysis of the genes that constitute this pathway in the network, we find that most genes are a form of the Class I and Class II type major histocompatibility complex (MHC). There exists enough evidence that MHC proteins in normal skeletal muscle fibers show lower expression levels, when compared to DMD [46]. Prior work also shows that for every MHC protein, the fold change for DMD muscle is greater than one [47], which represents a higher expression in DMD than in normal. Thus, we see that the algorithm, not only highlights the more enhanced pathways in the communities, but also identifies the lowly expressed pathways in the normal muscle. This evidence provides more confidence to the robustness of the communities detected. Table 10 shows a few genes from cell adhesion that are correlated to focal adhesion pathway.

## Methods

### Muscular Dystrophy: Dataset Description

We used the skeletal muscle gene expression data, *Series GSE6011* from the Gene Expression Omnibus [48]. The gene expression dataset consisted of measurements on probes for genes with a many-to-many mapping between probes and genes. In order to obtain one-to-one equivalence between the probes and genes, we perform a series of pre-processing steps, which are included in the Supporting Information (see Appendix S1). Table 11 summarizes the parameters of the pre-processed dataset.

**Table 11:** Summary of pre-processed GSE6011 dataset parameters.

Dataset	Number of Probes/Genes	Number of Experiments
Normal muscle	7685	13
DMD muscle	7685	23

### Derived interaction networks

We introduce the notion of an *interaction network* that is *derived* from an underlying gene expression dataset. This is one of the novel contributions in our paper. We consider a gene expression dataset 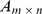 (consisting of measurements on 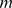
probes for genes across 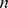 experiments) that has been pre-processed to represent one-on-one mappings between probes and genes. Let 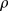
denote the *correlation matrix* for the dataset, containing the pairwise linear correlation coefficient between each pair of columns in the matrix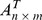, where 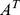 denotes the transpose of the matrix 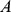 % 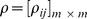



We define the *interaction network* for the dataset as an undirected network 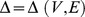, such that the set of vertices 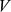
corresponds to the set of genes in the underlying dataset (i.e.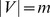) and the interactions between them are captured by the set of edges 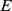 via an adjacency matrix as follows:
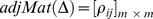


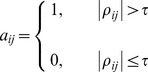



where 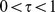 is a pre-defined threshold

Our intuition behind the definition of the interaction network was to capture the *inherent associations* between genes in a dataset, by using the correlation of expression measurements as a representative surrogate for the interactions between the underlying genes. In other words, the hypothesis is that a stronger correlation is likely to signify a stronger interaction between the genes exhibiting the correlation (modeled by the presence of an edge between the genes in the interaction network), while a weaker correlation is likely to correspond to a weaker interaction between the genes (modeled by the absence of an edge).

### Derived interaction networks for the GSE6011 Dataset

We generated the derived interaction networks for the pre-processed GSE6011 dataset for both the normal and DMD data. We used a threshold of 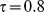 as the correlation cut-off, applying the guidelines from [40]. Hence, an edge was present between two genes in the generated interaction network if and only if the absolute value of correlation between those genes was greater than 0.8. We note that due to the post-processing steps described in the Supporting Information (see Appendix S1), the actual number of vertices considered for subsequent analysis in this paper is less than the initial number of vertices in the raw interaction networks generated for both normal and DMD data (summarized in Table 1).

### Pathway Analysis

Consequence of DMD pathology manifests in the state of muscle cells. The physiological state and cellular state of muscles are altered, involving concomitant changes in the expression of genes associated with the physiological function. In particular, gene expressions in DMD patients have the potential to provide information on distinguishing characteristics of pathology, relative to normal muscle (since altered gene expressions could aid in identification of functional communities). In this work, we have devised a novel approach to analyze human DMD patient gene expression data using a combination of techniques from linear algebra and network theory. Specifically, we posit that the correlation of gene expression data from DMD patients captures salient characteristics of pathology. Accordingly, we build the correlation network from the gene expression data for the normal and DMD muscles. Under the assumption that correlation implies mechanistic causality, we take the approach of community structure analysis, to identify functional communities from the correlation network, to display known functional and pathway mechanisms.

In this section, we present an analysis of the communities from the perspective of the pathways that the constituent genes represent. The goal is to understand the communities from derived interaction networks through functional analysis, since functions help elucidate alterations in pathological conditions [49]–[50]. Furthermore, we expect that the analysis of normal and DMD interaction networks from a pathway perspective is likely to yield more holistic insights into the correlation between functional and structural organization of the underlying genetic interactions.

**Figure 4:**
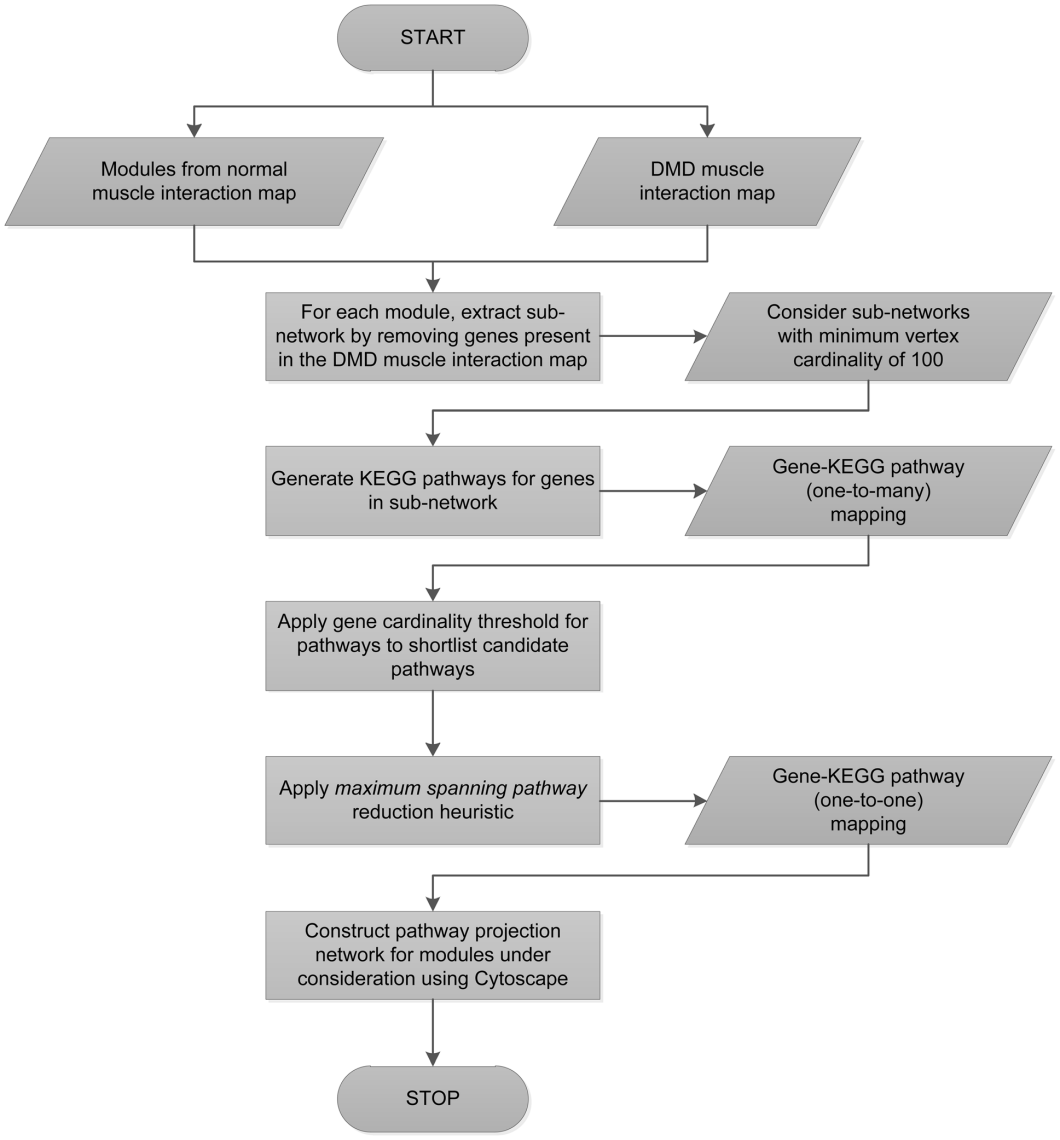
Schematic representation of transformation technique employed to generate PPNs. A schematic representation of the transformation technique that was employed to represent the communities from the perspective of the pathways that the constituent genes correspond to. This technique is described in detail in the Methods section.

We describe below, the transformation technique we employed to generate an equivalent network in terms of the constituent pathways for each community [also schematically presented in the flowchart in Figure 4]. We call this a *Pathway Projection Network* (PPN). For each community from the normal muscle interaction network, we extract a sub-network consisting of only those genes present in the normal muscle network and not in the DMD muscle network. From these sub-networks, we identify those that have a minimum vertex cardinality of 100 (we found four such candidates), and performed pathway analysis for these candidates using the KEGG mapper [51]–[52].

It is important to note that there is a one-to-many mapping between genes and pathways. Hence there are multiple pathway assignments that are possible for a given gene and this would lead to a combinatorial explosion in the number of pathway projection networks. To avoid this, we prune the space of gene-pathway mappings by employing a heuristic that we call the *maximum spanning pathway reduction heuristic*. This heuristic works as follows: From all candidate pathways that a gene from a sub-network belongs to, we choose that pathway **p** which maximizes the number of *other* genes spanning the sub-network which can also be assigned the pathway **p**.

We use Cytoscape to visualize the PPNs and these are shown in Figures 3A–3D (denoted as PPN1–PPN4). The PPNs 1–4 use the following convention. The pathway-nodes are color coded from Green to Red, with increasing degree of the node. This is a measure of connectivity between the pathways. A second attribute (pathway cardinality) defines the size of the node- a larger node signifying a larger pathway cardinality, which is the number of genes from the community that correspond to that pathway). Thus, strong connections between two large, red nodes imply a strong coupling between the set of genes in one pathway to the set that correspond to another.

From among the pathways represented in the PPNs, we are specifically interested in further analyzing pathways that are enhanced in each community and/or are known to be relevant to DMD from prior work [21]–[24], [32]–[38], [40]–[42]. These are summarized in Table 5. The pathway interactions analysis for the resultant PPNs is presented in the Results and Discussion section.

### Conclusion

In this paper, we have proposed a principled approach for transforming gene expression datasets into interaction networks, which serve as a useful representation for downstream analysis of pathology. Furthermore, we have illustrated the utility of community structure analysis applied to the interaction networks, as a sound computational technique for gaining insights about the underlying topology and function. We have leveraged this approach to study the characteristics of normal and DMD human skeletal muscle tissues, in terms of functional communities. In addition to providing a topological perspective on the differential regulation of transcripts between normal and DMD skeletal muscle, the derived communities provide extensive information on functional pathways and their association with pathology. Not only does our analysis provide clear evidence of the role of altered metabolic, calcium signaling and cytoskeletal remodeling pathways in DMD, but also identifies novel cross-talk between them. We believe that our work provides the steps for biomarker identification, as well as systems level information for therapy of the DMD skeletal muscle.

## Supporting Information

Appendix S1 **GSE6011 dataset description and post-processing steps on the derived interaction networks.**
(DOCX)

Figure S1 **Pathway Projection Network 1.** Pathway Projection Network from the 1^st^ dominant topological community (in terms of size). This PPN represents enhancement of metabolic pathways. We also observe coupling between metabolic pathways and other pathways represented in the same community, such as regulation of actin cytoskeleton.
(TIF)

Figure S2 **Pathway Projection Network 2.** Pathway Projection Network from the 2^nd^ dominant topological community (in terms of size). This PPN represents enhancement of metabolic pathways. We also observe coupling between metabolic pathways and other pathways represented in the same community, such as pathways in cancer.
(TIF)

Figure S3 **Pathway Projection Network 3.** Pathway Projection Network from the 3^rd^ dominant topological community (in terms of size). This PPN represents enhancement of metabolic pathways. We also observe coupling between metabolic pathways and other pathways represented in the same community, such as calcium signaling pathway.
(TIF)

Figure S4 **Pathway Projection Network 4.** Pathway Projection Network from the 4^th^ dominant topological community (in terms of size). This PPN represents enhancement of focal adhesion pathways and regulation of actin cytoskeleton. We also observe coupling between focal adhesion pathways and other pathways represented in the same community, such as regulation of actin cytoskeleton and cell adhesion molecules.
(TIF)

Figure S5 **Pathway Projection Network 5.** Pathway Projection Network from the 5^th^ dominant topological community (in terms of size). This PPN represents enhancement of metabolic pathways.
(TIF)

Figure S6 **Pathway Projection Network 6.** Pathway Projection Network from the 6^th^ dominant topological community (in terms of size). This PPN represents enhancement of metabolic pathways and aminoacyl.
(TIF)

Figure S7 **Pathway Projection Network 7.** Pathway Projection Network from the 7^th^ dominant topological community (in terms of size). This PPN represents enhancement of metabolic pathways. We also observe coupling between metabolic pathways and other pathways represented in the same community, such as the signaling pathways.
(TIF)

Figure S8 **Pathway Projection Network 8.** Pathway Projection Network from the 8^th^ dominant topological community (in terms of size). This PPN represents enhancement of pathways in cancer.
(TIF)

Figure S9 **Pathway Projection Network 9.** Pathway Projection Network from the 9^th^ dominant topological community (in terms of size). This PPN represents enhancement of metabolic pathwayys and arrhythmogenic right ventricular cardiomyopathy.
(TIF)

Figure S10 **Pathway Projection Network 10.** Pathway Projection Network from the 10^th^ dominant topological community (in terms of size). This PPN represents enhancement of metabolic pathways and MAPK signaling pathway.
(TIF)

Figure S11 **Pathway Projection Network 11.** Pathway Projection Network from the 11^th^ dominant topological community (in terms of size). This PPN represents enhancement of metabolic pathways and Huntington's disease.
(TIF)
