# Supplementary material for: Community Structure Analysis of Gene Interaction Networks in Duchenne Muscular Dystrophy
Source: PLoS One. 2013 Jun 19;8(6):e67237. doi: 10.1371/journal.pone.0067237 (PMC3686745; doi:10.1371/journal.pone.0067237)
Supplement: Appendix S1 — GSE6011 dataset description and post-processing steps on the derived interaction networks. (DOCX) [file pone.0067237.s001.docx]

**Appendix S1**

**GSE6011: Dataset description**

We used the skeletal muscle gene expression data, *Series GSE6011* from the Gene Expression Omnibus [Pescatori et al., 2007]. The gene expression dataset consisted of measurements on probes for genes with a many-to-many mapping between probes and genes. The number of experiments for normal and dystrophy data were 13 and 23 respectively. The data was Mas 5 normalized and sample outliers were removed. Additionally, the probes with no entrez IDs were also eliminated and the many-to-many mappings were accounted for, using the following procedure. The correspondence between multiple probes for a single gene was collapsed [Miller et al., 2011], by taking the *mean* of the expression if there were two entries of probes corresponding to one gene; and by taking the *maximum influence* shown in an expression for a particular probe if more than 2 probes represented one gene. The single probes that corresponded to multiple genes were eliminated since we were interested in the correlation network built from the data and thus were keen on exploring only unique gene expressions.

**Derived interaction networks for the GSE6011 Dataset**

We performed the following post-processing steps on the derived interaction networks in order to make them more amenable for effective analysis, from a descriptive statistics standpoint and from a community structure standpoint.

Firstly, we removed the self-loops from the derived interaction networks i.e. all the diagonal elements in the adjacency matrix were assigned a value 0 (these self-loops resulted from the self-correlation values of $\rho_{ii}=1$ in the corresponding correlation matrices).


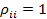


Secondly, it is important to note that there were some genes which did not share a good correlation with *any* other gene (in both the normal and DMD data) i.e. their correlation score$\left|\rho\right|$was below the threshold of 0.8. Hence, these genes had no edges connected to them in the interaction network and were represented as isolated vertices in the adjacency matrix. Such “orphaned genes” were also removed from the interaction network.


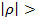


**References**

Pescatori M, Broccolini A, Minetti C, Bertini E et al. Gene expression profiling in the early phases of DMD: a constant molecular signature characterizes DMD muscle from early postnatal life throughout disease progression. FASEB J 2007 (4):1210-26

Miller JA, Cai C, Langfelder P, Geschwind DH, Kurian SM, Salomon DR, Horvath S: Strategies for aggregating gene expression data: The collapseRows R function. BMC Bioinformatics 2011, 12:322
