# Supplementary figures and images for: Community Structure Analysis of Gene Interaction Networks in Duchenne Muscular Dystrophy
Source: PLoS One. 2013 Jun 19;8(6):e67237. doi: 10.1371/journal.pone.0067237 (PMC3686745; doi:10.1371/journal.pone.0067237)

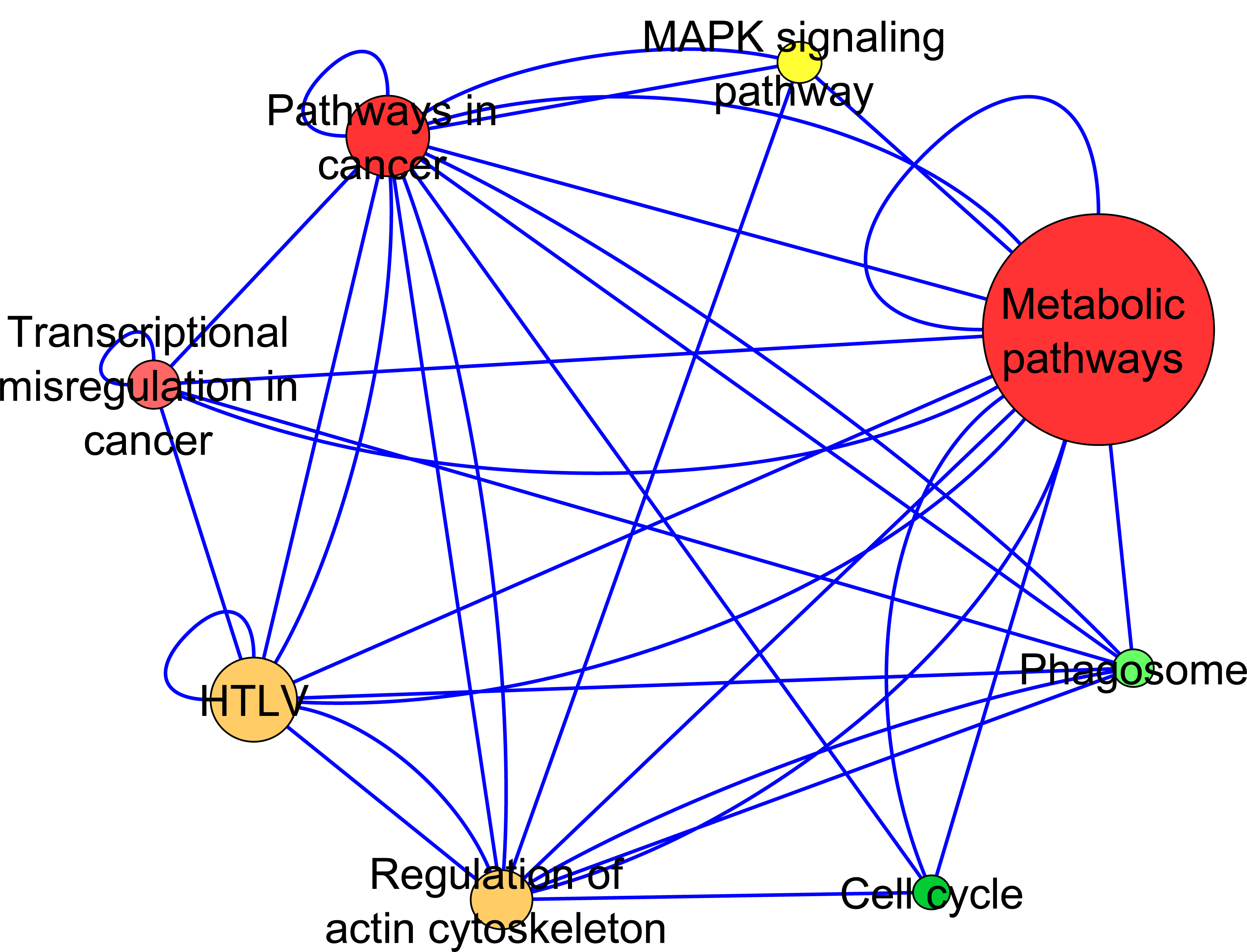

Supplement: Figure S1 — Pathway Projection Network 1. Pathway Projection Network from the 1st dominant topological community (in terms of size). This PPN represents enhancement of metabolic pathways. We also observe coupling between metabolic pathways and other pathways represented in the same community, such as regulation of actin cytoskeleton. (TIF) [file pone.0067237.s002.tif]

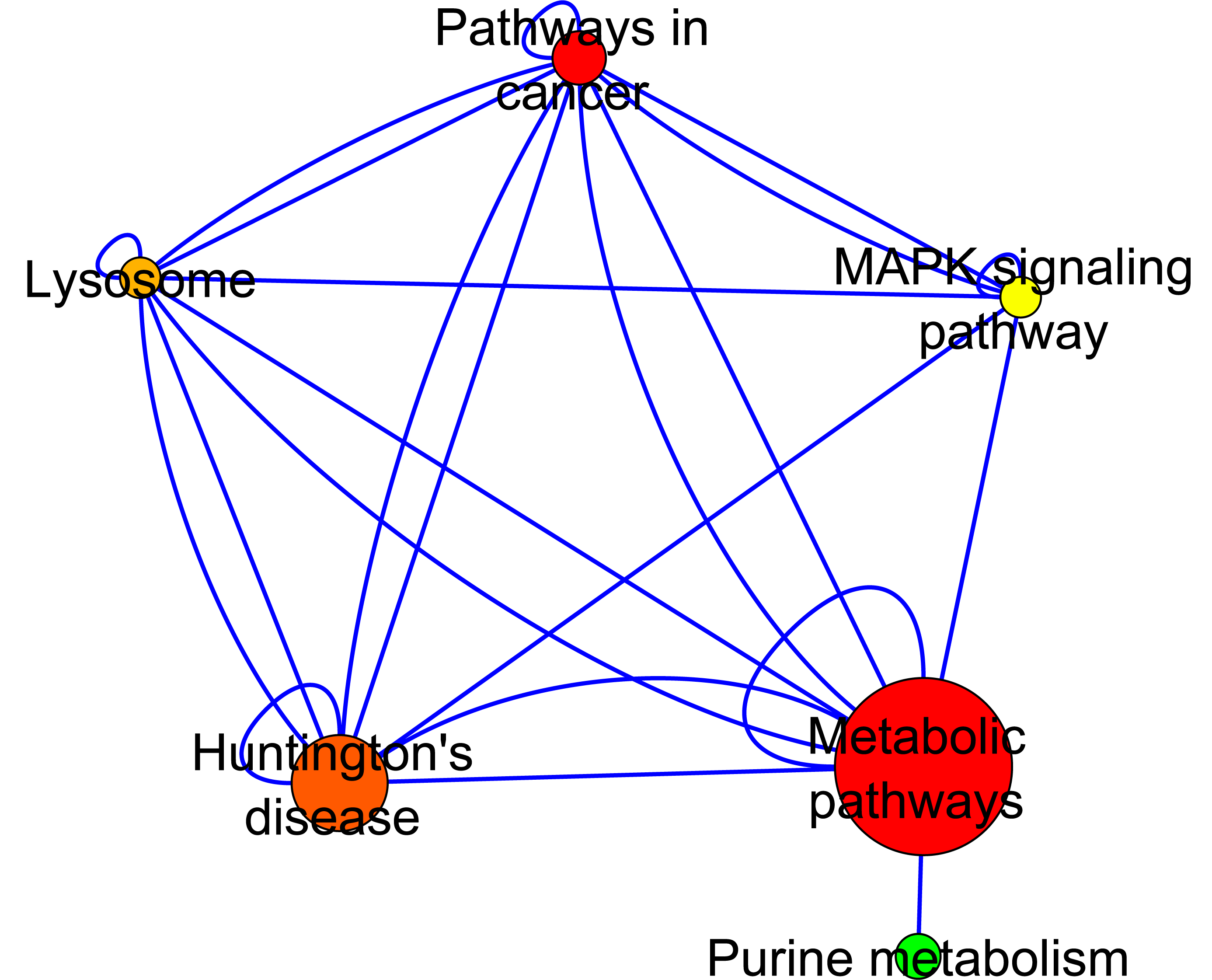

Supplement: Figure S2 — Pathway Projection Network 2. Pathway Projection Network from the 2nd dominant topological community (in terms of size). This PPN represents enhancement of metabolic pathways. We also observe coupling between metabolic pathways and other pathways represented in the same community, such as pathways in cancer. (TIF) [file pone.0067237.s003.tif]

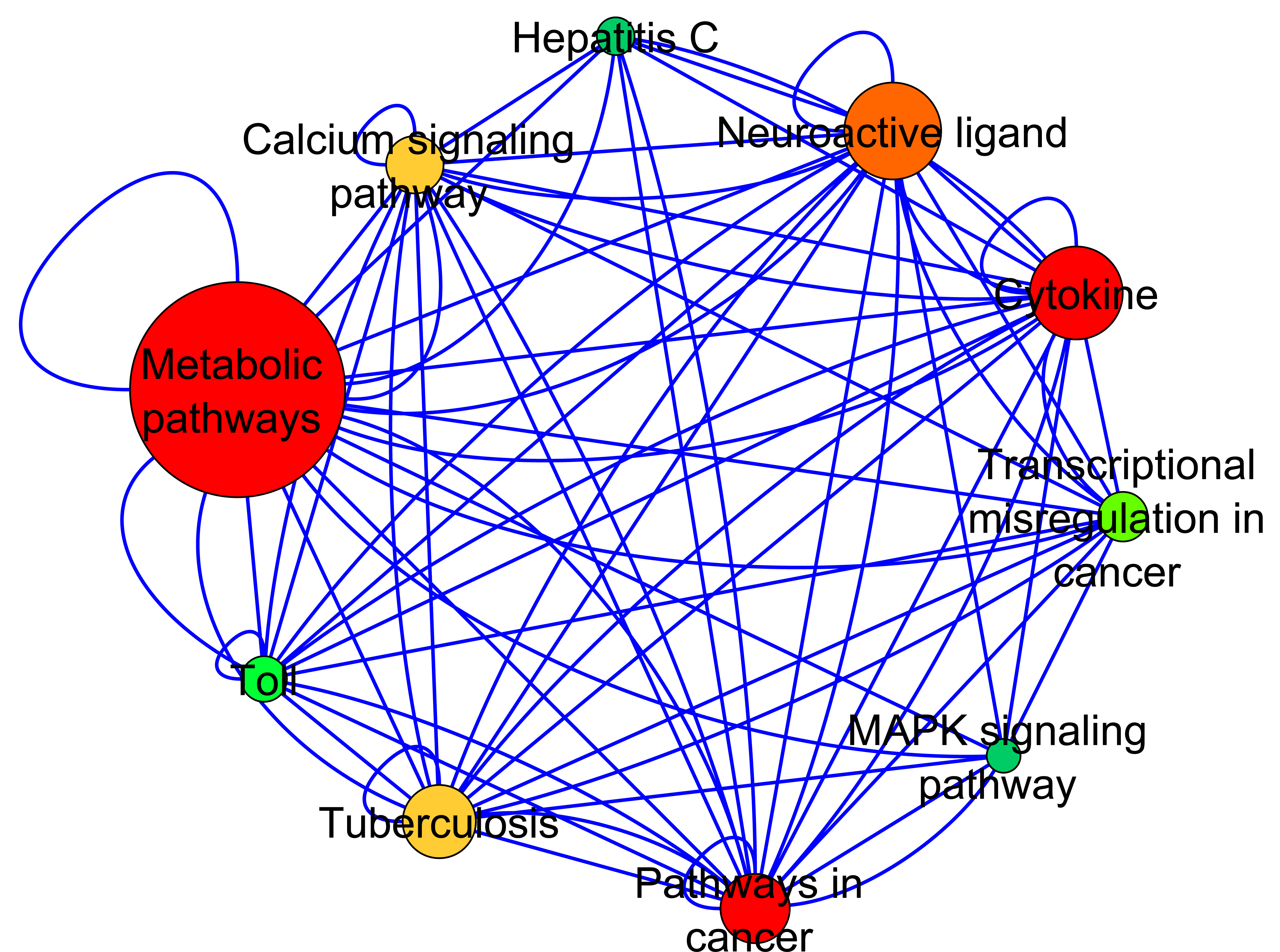

Supplement: Figure S3 — Pathway Projection Network 3. Pathway Projection Network from the 3rd dominant topological community (in terms of size). This PPN represents enhancement of metabolic pathways. We also observe coupling between metabolic pathways and other pathways represented in the same community, such as calcium signaling pathway. (TIF) [file pone.0067237.s004.tif]

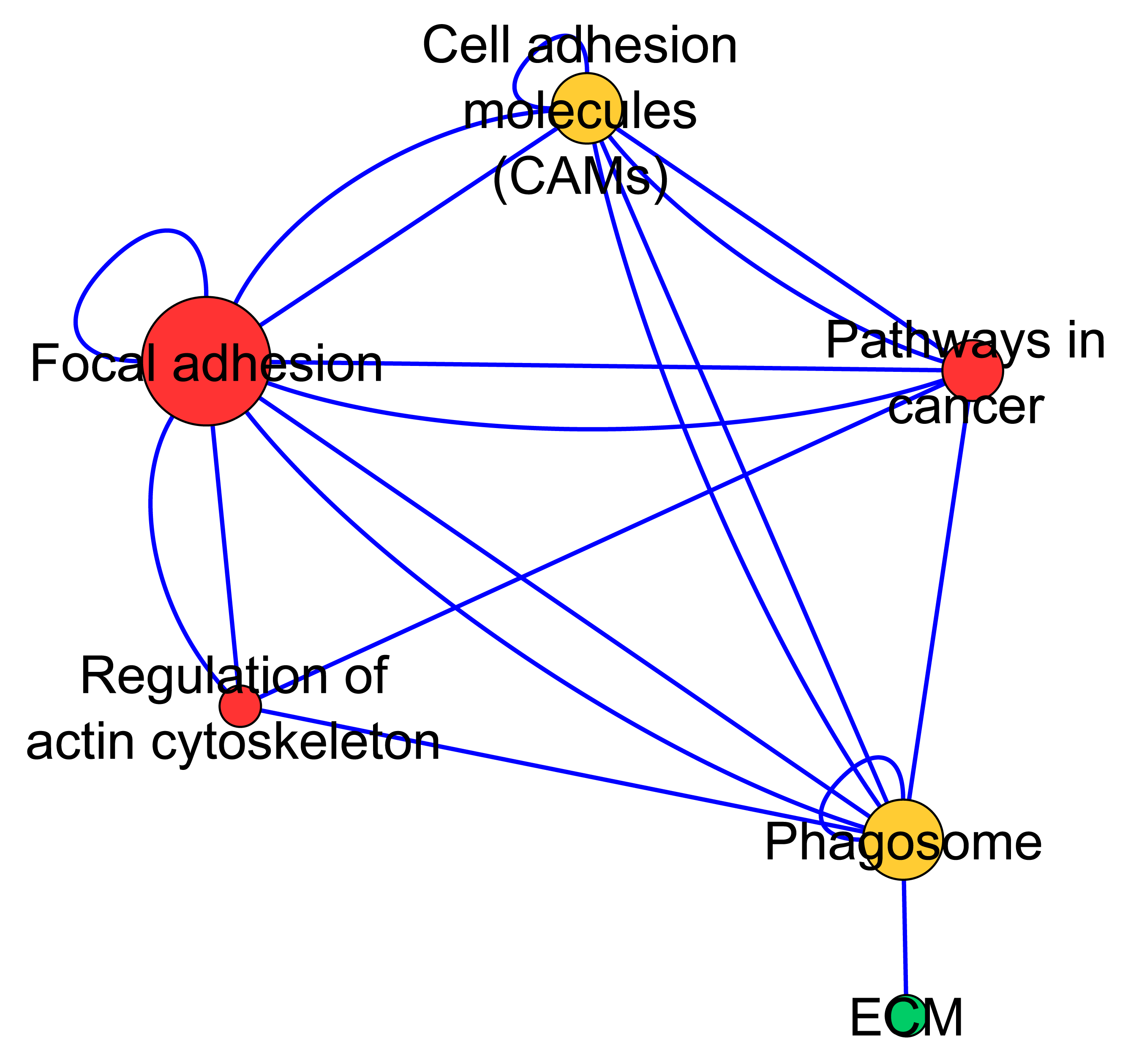

Supplement: Figure S4 — Pathway Projection Network 4. Pathway Projection Network from the 4th dominant topological community (in terms of size). This PPN represents enhancement of focal adhesion pathways and regulation of actin cytoskeleton. We also observe coupling between focal adhesion pathways and other pathways represented in the same community, such as regulation of actin cytoskeleton and cell adhesion molecules. (TIF) [file pone.0067237.s005.tif]

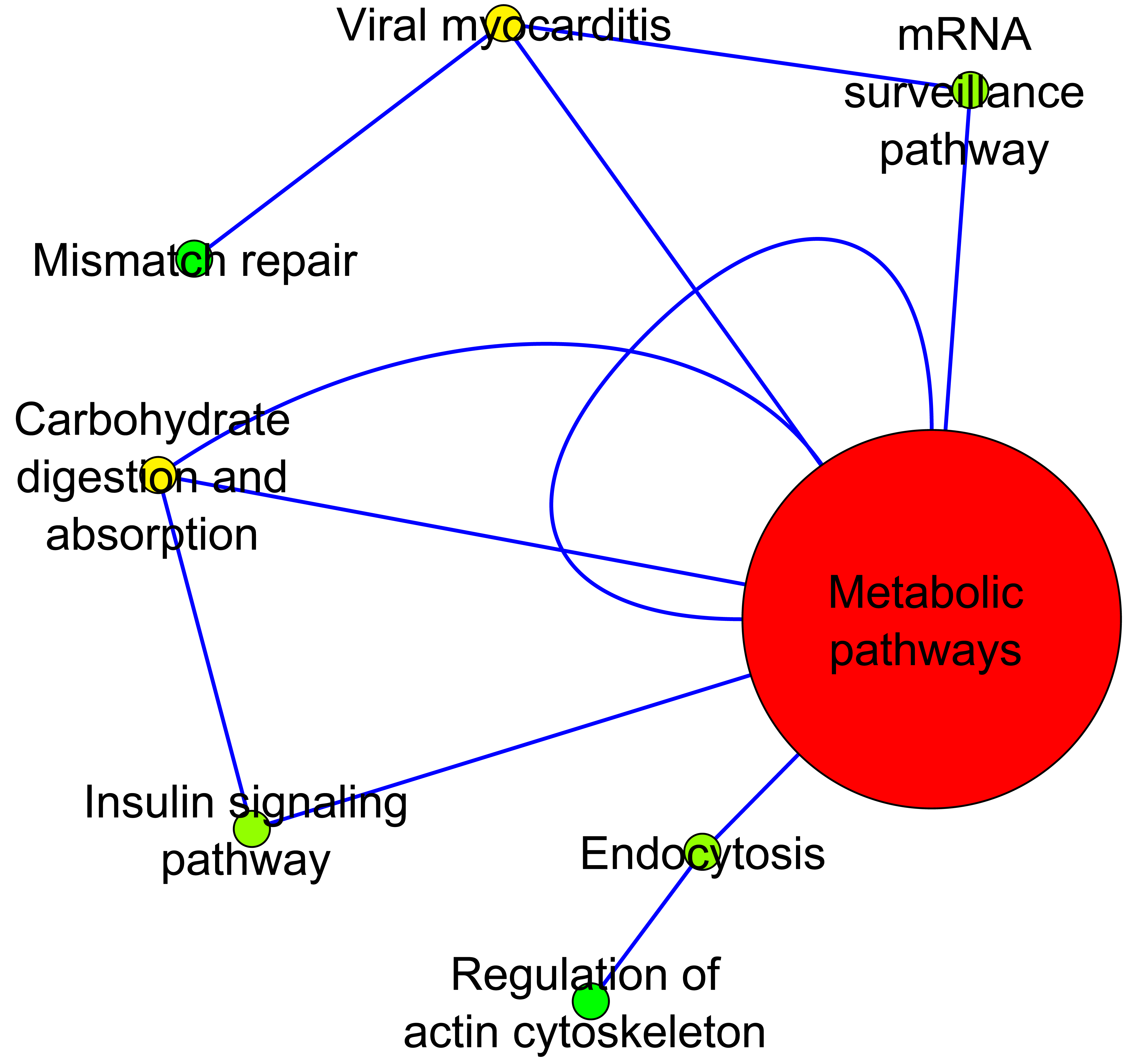

Supplement: Figure S5 — Pathway Projection Network 5. Pathway Projection Network from the 5th dominant topological community (in terms of size). This PPN represents enhancement of metabolic pathways. (TIF) [file pone.0067237.s006.tif]

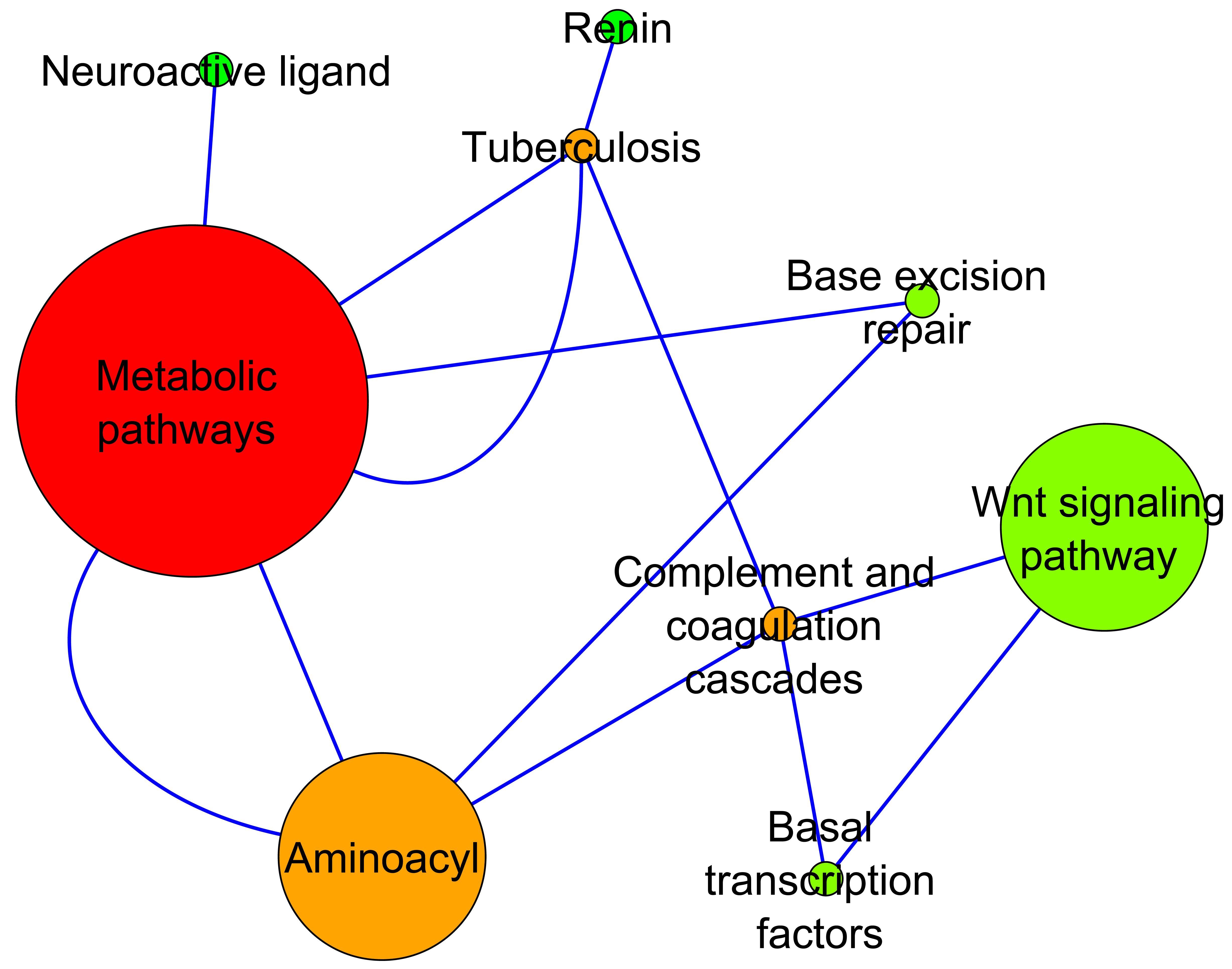

Supplement: Figure S6 — Pathway Projection Network 6. Pathway Projection Network from the 6th dominant topological community (in terms of size). This PPN represents enhancement of metabolic pathways and aminoacyl. (TIF) [file pone.0067237.s007.tif]

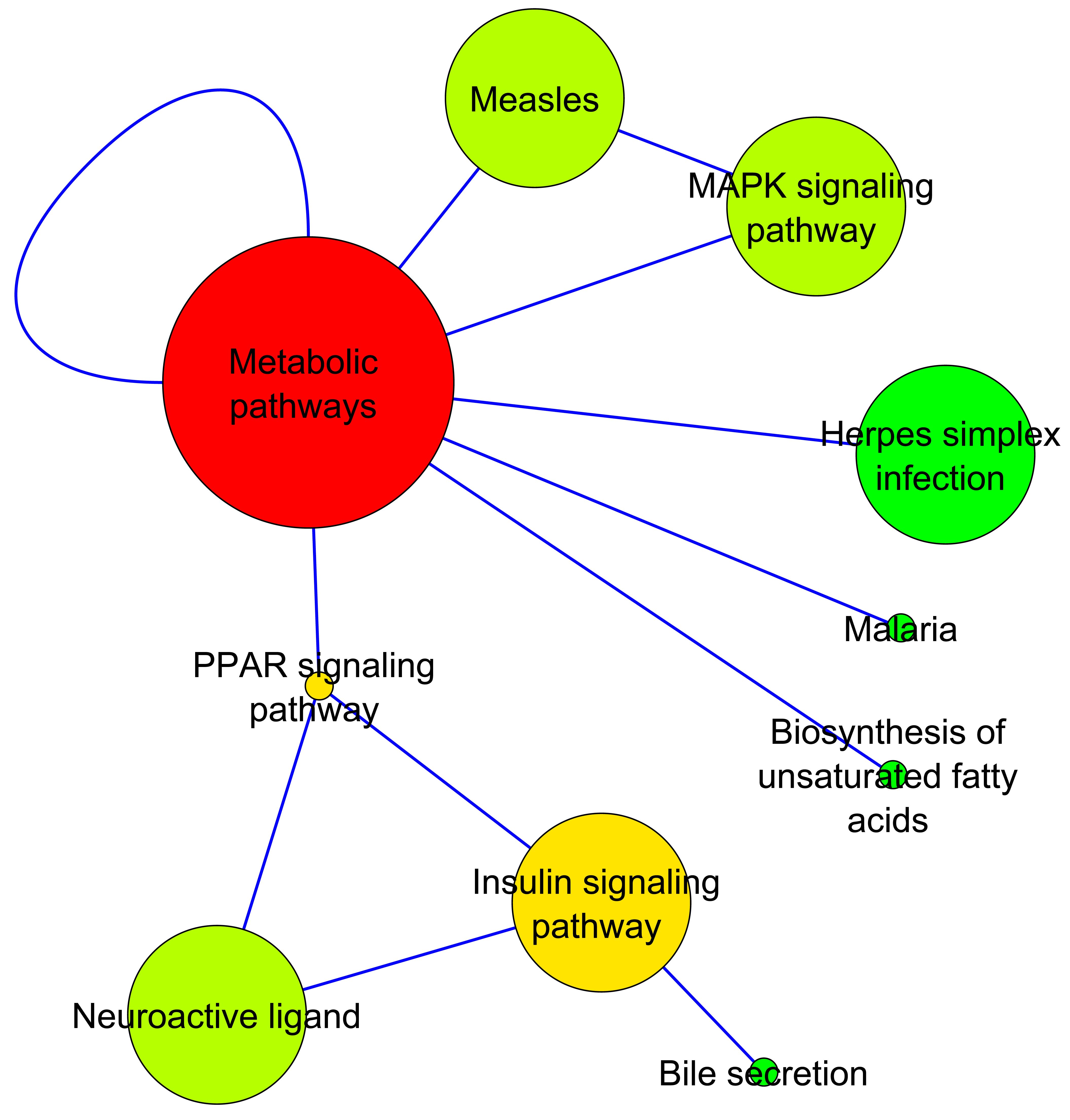

Supplement: Figure S7 — Pathway Projection Network 7. Pathway Projection Network from the 7th dominant topological community (in terms of size). This PPN represents enhancement of metabolic pathways. We also observe coupling between metabolic pathways and other pathways represented in the same community, such as the signaling pathways. (TIF) [file pone.0067237.s008.tif]

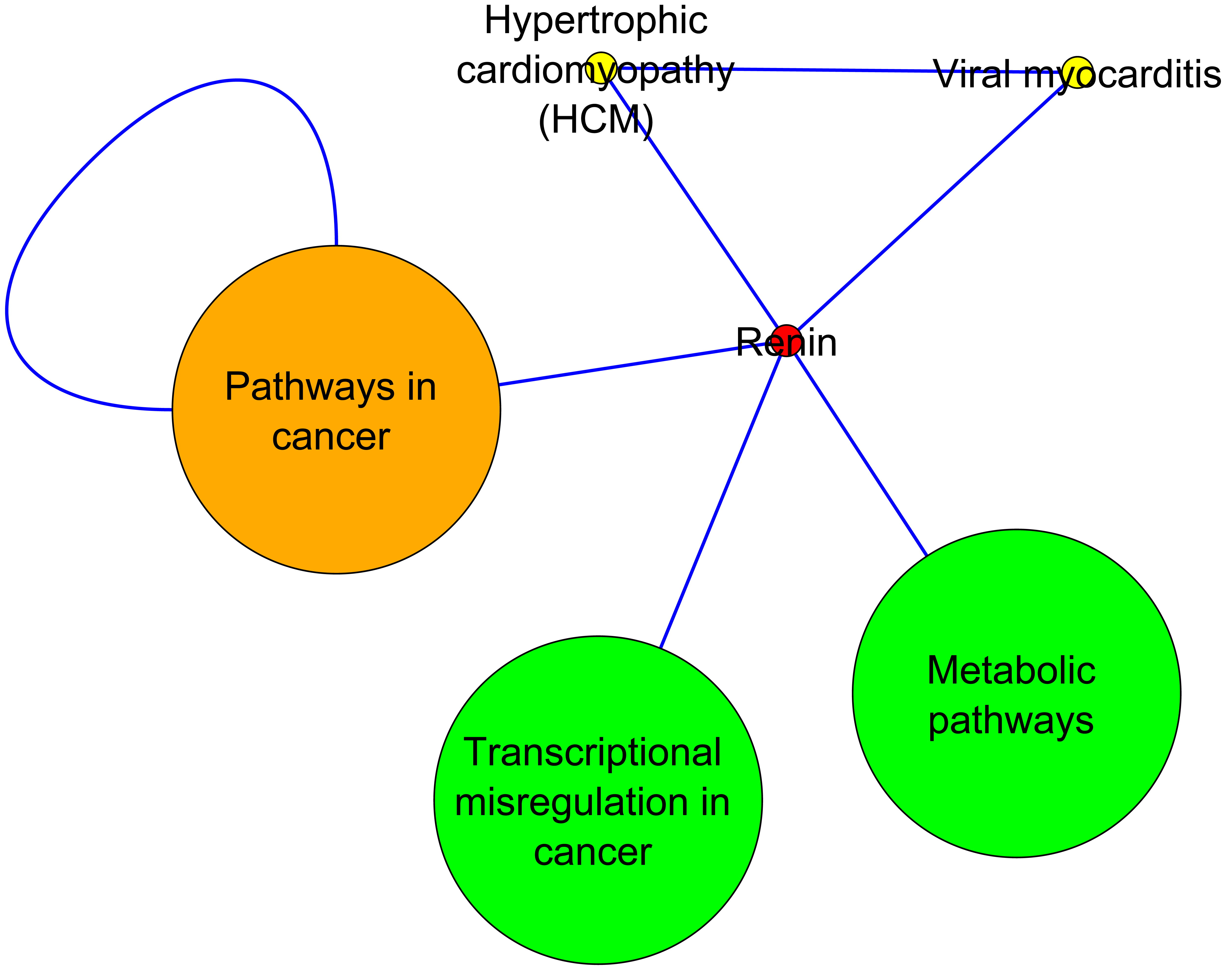

Supplement: Figure S8 — Pathway Projection Network 8. Pathway Projection Network from the 8th dominant topological community (in terms of size). This PPN represents enhancement of pathways in cancer. (TIF) [file pone.0067237.s009.tif]

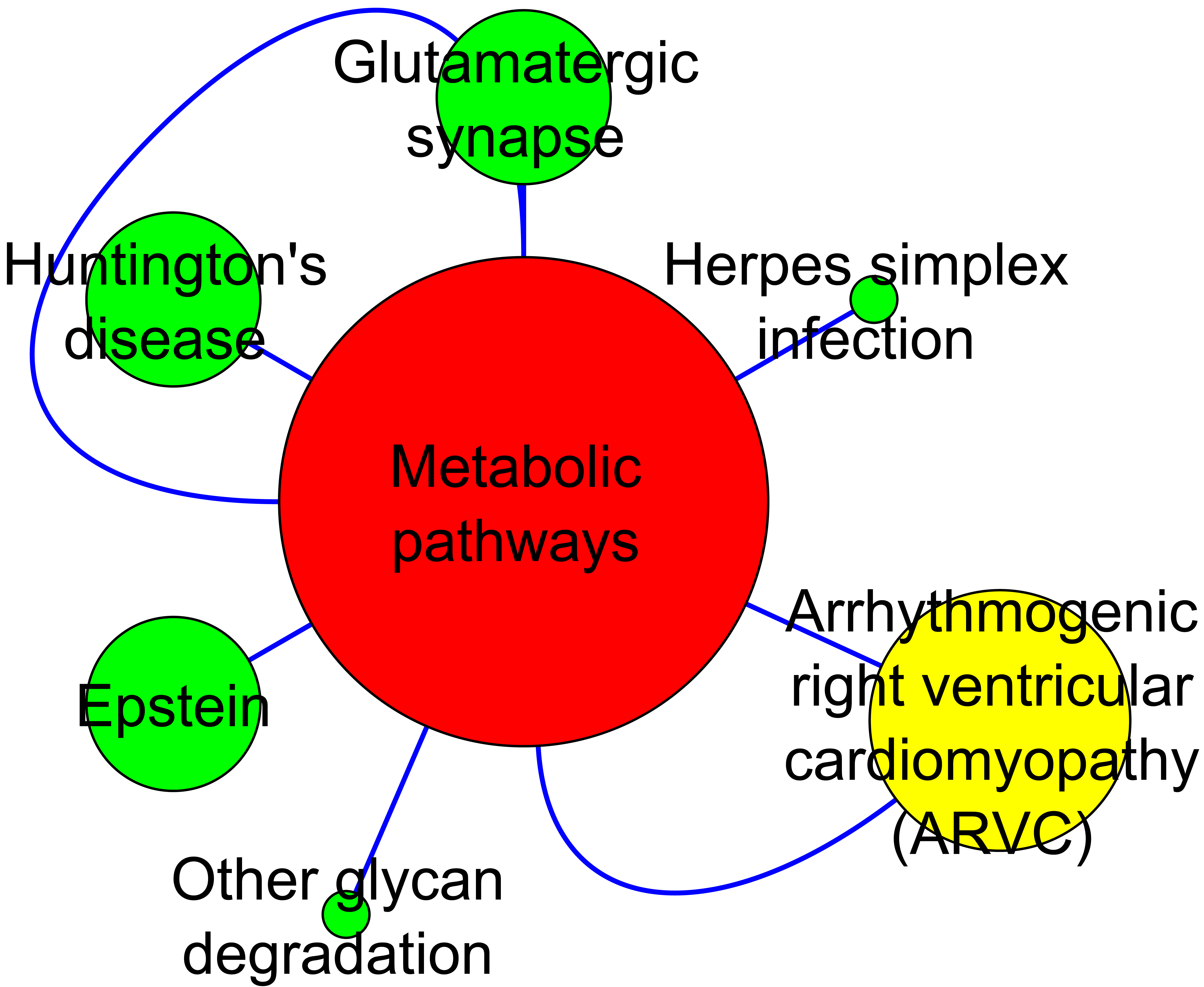

Supplement: Figure S9 — Pathway Projection Network 9. Pathway Projection Network from the 9th dominant topological community (in terms of size). This PPN represents enhancement of metabolic pathwayys and arrhythmogenic right ventricular cardiomyopathy. (TIF) [file pone.0067237.s010.tif]

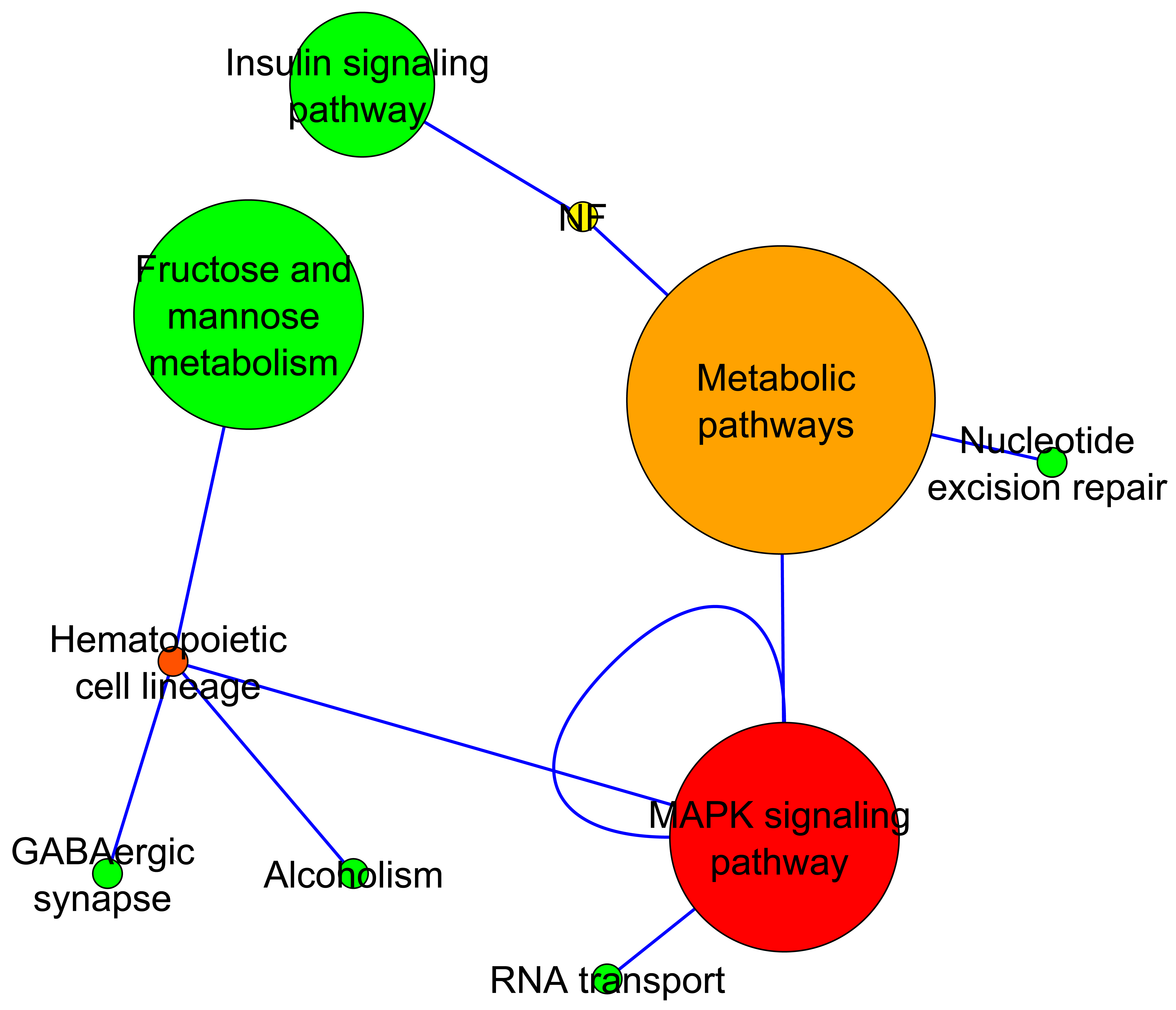

Supplement: Figure S10 — Pathway Projection Network 10. Pathway Projection Network from the 10th dominant topological community (in terms of size). This PPN represents enhancement of metabolic pathways and MAPK signaling pathway. (TIF) [file pone.0067237.s011.tif]

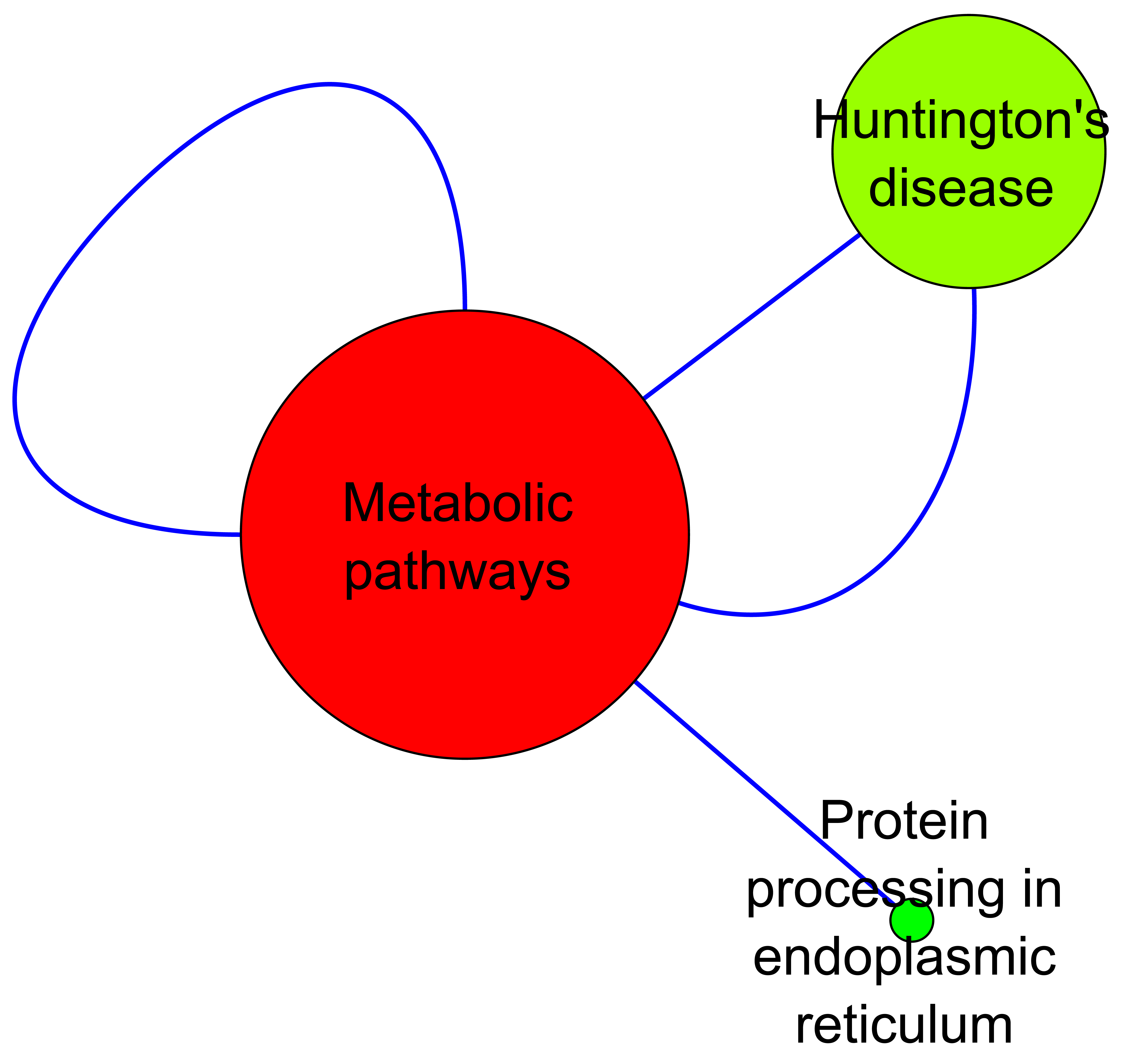

Supplement: Figure S11 — Pathway Projection Network 11. Pathway Projection Network from the 11th dominant topological community (in terms of size). This PPN represents enhancement of metabolic pathways and Huntington's disease. (TIF) [file pone.0067237.s012.tif]
